# Downregulation of SPTAN1 is related to MLH1 deficiency and metastasis in colorectal cancer

**DOI:** 10.1371/journal.pone.0213411

**Published:** 2019-03-11

**Authors:** Anne Ackermann, Christopher Schrecker, Dimitra Bon, Nicolaus Friedrichs, Katrin Bankov, Peter Wild, Guido Plotz, Stefan Zeuzem, Eva Herrmann, Martin-Leo Hansmann, Angela Brieger

**Affiliations:** 1 Medical Clinic I, Biomedical Research Laboratory, University Clinic Frankfurt, Frankfurt am Main, Germany; 2 Institute for Biostatistics and Mathematical Modelling, Goethe University, Frankfurt am Main, Germany; 3 Institute of Pathology, University of Cologne Medical School, Cologne, Germany; 4 Dr. Senckenberg Institute of Pathology, University Hospital Frankfurt, Frankfurt am Main, Germany; Sapporo Ika Daigaku, JAPAN

## Abstract

**Introduction:**

Colorectal cancers (CRCs) deficient in the DNA mismatch repair protein MutL homolog 1 (MLH1) display distinct clinicopathological features and require a different therapeutic approach compared to CRCs with MLH1 proficiency. However, the molecular basis of this fundamental difference remains elusive. Here, we report that MLH1-deficient CRCs exhibit reduced levels of the cytoskeletal scaffolding protein non-erythroid spectrin αII (SPTAN1), and that tumor progression and metastasis of CRCs correlate with SPTAN1 levels.

**Methods and results:**

To investigate the link between MLH1 and SPTAN1 in cancer progression, a cohort of 189 patients with CRC was analyzed by immunohistochemistry. Compared with the surrounding normal mucosa, SPTAN1 expression was reduced in MLH1-deficient CRCs, whereas MLH1-proficient CRCs showed a significant upregulation of SPTAN1. Overall, we identified a strong correlation between MLH1 status and SPTAN1 expression. When comparing TNM classification and SPTAN1 levels, we found higher SPTAN1 levels in stage I CRCs, while stages II to IV showed a gradual reduction of SPTAN1 expression. In addition, SPTAN1 expression was lower in metastatic compared with non-metastatic CRCs. Knockdown of SPTAN1 in CRC cell lines demonstrated decreased cell viability, impaired cellular mobility and reduced cell-cell contact formation, indicating that SPTAN1 plays an important role in cell growth and cell attachment. The observed weakened cell-cell contact of SPTAN1 knockdown cells might indicate that tumor cells expressing low levels of SPTAN1 detach from their primary tumor and metastasize more easily.

**Conclusion:**

Taken together, we demonstrate that MLH1 deficiency, low SPTAN1 expression, and tumor progression and metastasis are in close relation. We conclude that SPTAN1 is a candidate molecule explaining the tumor progression and metastasis of MLH1-deficient CRCs. The detailed analysis of SPTAN1 is now mandatory to substantiate its relevance and its potential value as a candidate protein for targeted therapy, and as a predictive marker of cancer aggressiveness.

## Introduction

Colorectal cancer (CRC) is one of the three most commonly diagnosed tumors worldwide and the fourth most common cause of cancer deaths. It is estimated that the global incidence of and mortality from CRC will increase in the next 10–20 years to more than 2.2 million new cases and 1.1 million cancer deaths annually [1].

CRC is a heterogeneous malignant tumor with regard to molecular pathogenesis and genetic instability. The majority of CRCs display chromosomal instability and follow the classical adenoma-carcinoma sequence of tumor progression [2]. About 15% of CRCs show loss of DNA mismatch repair (MMR) and a microsatellite instability-high (MSI-H) phenotype [3]. 20% of these MSI-H CRCs are due to germline mutations in one of the MMR genes (most often *MutL homolog 1 (MLH1)* or *MutS homolog 2* (*MSH2*)) and are associated with a hereditary form of CRC called Lynch syndrome [4]. The remainder are of sporadic origin and caused by hypermethylation of the promoter of the *MLH1* gene [5], which is associated with a V600E missense mutation in the *BRAF* oncogene [6]. Therefore, MMR deficiency in CRCs is most often produced by loss of the MMR protein MLH1.

MSI-H CRCs differ markedly from sporadic CRCs in that they are usually associated with proximal tumor localization, poor differentiation, mucinous histology and boast dense local lymphocytic infiltrates [7]. In addition, MSI-H CRCs are most often diagnosed at an earlier stage compared with their MMR-proficient counterparts [8, 9]. In early-stage CRC, MSI-H is associated with a better prognosis and low aggressiveness [10], whereas MSI-H metastatic disease seems to confer a negative prognosis [11, 12]. The molecular explanation for these contradictory outcomes remains elusive. Given that cytoskeletal reorganization is a central feature of malignant transformation, elucidating the interactions between the MMR machinery and the cytoskeleton appears to be a reasonable approach [13]. Indeed, the MLH1 protein has been shown to interact with a number of cytoskeletal scaffolding proteins, namely non-erythroid spectrin αII (SPTAN1), Thymosin beta 4, Actin gamma, Cathepsin B, Annexin A2 and Desmin [14–16]. In addition, we previously reported that MLH1-deficient cell lines exhibit reduced levels of SPTAN1, leading to decreased migratory ability of those cells [15]. SPTAN1 is an important cytoskeletal scaffolding protein involved in a number of fundamental cellular processes including cell adhesion, cell-cell contact, and apoptosis [17–21]. Increased SPTAN1 expression has been described in various tumor entities including CRC, and appears to be related to tumor progression and invasion. Thus, SPTAN1 may serve as a potential biomarker for tumor aggressiveness.

In the current study, we analyzed the connection between MLH1 and SPTAN1 expression in a cohort of 189 patients with CRC. Furthermore, the influence of SPTAN1 reduction on cell viability, cell motility, and cell-cell contact was determined *in vitro* using three different CRC cell culture models.

## Materials and methods

### Patients

Paraffin-embedded tissue samples from 189 patients with surgically resected, well-characterized colorectal tumors, along with samples of the corresponding adjacent normal colonic mucosa, were selected for immunohistochemical analysis. Patients were divided into two groups: those with MLH1-deficient tumors and those with MLH1-proficient tumors. All patients included in the study underwent bowel resection with curative intent. Individuals with prior exposure to neoadjuvant chemoradiotherapy were excluded from the study, in order to avoid interference from cytoreductive therapies that may conceivably alter tumor genetics. Characteristics of the individual tissue specimens are summarized in the S1 Table. Basic clinical characteristics such as gender, age at diagnosis, tumor localization, year of diagnosis and surgery, tumor classification, metastases and tumor stage are listed in Table 1.

**Table 1:** Basic clinical characteristics of the cohort.

		All (n = 189)	MLH1 Positive (n = 162)	MLH1 Negative (n = 27)^1^	p-value^2^
Gender					
Female	n (%)	92 (48.7%)	75 (46.3%)	17 (63.0%)	0.145
Male	n (%)	97 (51.3%)	87 (53.7%)	10 (37.0%)	
Age at diagnosis	mean (sem)	68.4 (13.4)	68.0 (1.0)	65.8 (3.2)	> 0.20
Localization					
Distal	n (%)	67 (37.9%)	62 (40.0%)	5 (22.7%)	0.0103
Proximal	n (%)	110 (62.1%)	93 (60.0%)	17 (77.3%)	
Year of diagnosis and surgery					
before 2011	n (%)	3 (1.6%)	1 (0.6%)	2 (7.4%)	0.122
2011	n (%)	14 (7.4%)	14 (8.6%)	0 (0.0%)	
2012	n (%)	34 (18.0%)	31 (19.1%)	3 (11.1%)	
2013	n (%)	42 (22.2%)	37 (22.8%)	5 (18.5%)	
2014	n (%)	37 (19.6%)	31 (19.1%)	6 (22.2%)	
2015	n (%)	25 (13.2%)	21 (13.0%)	4 (14.8%)	
2016	n (%)	34 (18.0%)	27 (16.8%)	7 (26.0%)	
Tumor					
pT1/pT1a	n (%)	12 (6.3%)	10 (6.2%)	2 (7.4%)	0.172
pT2	n (%)	34 (18.0%)	31 (19.1%)	3 (11.1%)	
pT3	n (%)	113 (59.8%)	99 (61.1%)	14 (51.9%)	
pT4/pT4a/pT4b	n (%)	30 (15.9%)	22 (13.6%)	8 (29.6%)	
Metastases					
M0	n (%)	145 (76.7%)	124 (76.5%)	21 (77.8%)	> 0.20
M1	n (%)	17 (9.0%)	15 (9.3%)	2 (7.4%)	
M1a	n (%)	15 (7.9%)	13 (8.0%)	2 (7.4%)	
M1b	n (%)	12 (6.4%)	10 (6.2%)	2 (7.4%)	
Stage					
I	n (%)	38 (20.1%)	32 (19.8%)	6 (22.2%)	0.173
II/IIA/IIB/IIC	n (%)	58 (30.7%)	47 (29.0%)	11 (40.7%)	
III/IIIA/IIIB/IIIC	n (%)	52 (27.5%)	49 (30.2%)	3 (11.1%)	
IV/IVA/IVB	n (%)	41 (21.7%)	34 (21.0%)	7 (26.0%)	

^1^ 15 (55.6%) of the 27 MLH1-deficient CRCs were *BRAF* wt, and 12 (44.4%) showed mutated *BRAF* V600E. MLH1-deficient *BRAF* wt CRCs showed no difference in SPTAN1 expression compared with MLH1-deficient *BRAF* V600E CRCs (p = 0.958) (see S1 Table).

^2^p-values from comparisons between MLH1-positive and MLH1-negative patients, using Student’s t-test or Fisher’s exact test as appropriate.

Expression levels of both MLH1 and SPTAN1 were determined by immunohistochemistry for every tumor and adjacent non-malignant tissue.

The study was approved by the local ethics committee of the University Hospital Frankfurt, and all patients gave written informed consent.

### Cell lines, antibodies and plasmids

Colorectal adenocarcinoma Caco-2 cells (ATCC HTB-37), SW480 primary colon cancer cells (CCL-228) and their metastasized variant SW620 (CCL-227), derived from a lymph node metastasis, were purchased from the American Type Culture Collection (Rockville, MD), and HEK293T cells were obtained from Dr. Kurt Ballmer (Paul Scherrer Institute, Villigen, Switzerland). All cell lines were grown in DMEM (Dulbecco’s Modified Eagle Medium, Gibco, USA) with 10% FCS (Sigma-Aldrich, USA) and 1% Penicillin-Streptomycin (Sigma-Aldrich, USA). The cells were tested frequently for mycoplasma and characterized in April 2018 (Caco-2) and in June 2018 (HEK293T, SW480 and SW620) by STR profiling, as indicated by the DSMZ online catalogue [22]. STR profiling of the 8 STR loci was performed as recently described [23].

The following antibodies were used: anti-SPTAN1 (C-11, Santa Cruz Biotechnology, USA), anti-MLH1 (G168-728, BD Biosciences, USA), anti-beta-Actin (clone AC-15, Sigma-Aldrich, USA), anti-SPTAN1 (MAB1622, Millipore, USA), IRDye 680LT Goat anti-Mouse (LiCor Biosciences, USA), anti-ZO-1 (40–2200, Invitrogen, USA), and goat anti-rabbit Alexa Fluor 488 (A-11034, Invitrogen, USA).

All vectors for lentiviral transduction were purchased from Sigma-Aldrich, USA. Two different shSPTAN1 plasmids were used (MISSION shRNA TRCN0000053668 and MISSION shRNA TRCN0000053669). The MISSION pLKO.1-puro vector (SHC002V) was used as a control.

### Transduction with SPTAN1-shRNA

Caco-2, SW480 and SW620 cells were transduced with lentivirus encoding interfering nucleic acid molecules, according to the manufacturer’s protocol (Sigma-Aldrich, Mission). Cells were plated at a density of 1 × 10^5^ cells per well and transduced with 3μg of two different shRNAs targeting SPTAN1, delivered through a viral vector. As a control, Caco-2, SW480 and SW620 cells were also transduced with the same amount of viral vector containing non-mammalian shRNA. Transduced cells were selected for in puromycin-containing (5 μg/ml) cell culture medium.

### Western blot analysis

SPTAN1 expression was determined by Western blotting using anti-SPTAN1 antibody (MAB1622, 1:1000 dilution). Proteins were separated on 10% polyacrylamide gels, followed by Western blotting on nitrocellulose membranes and antibody detection using standard procedures. Fluorescent-labeled secondary antibodies (anti-mouse 680LT from LiCor Bioscience, USA) were used to detect signals in a FLA-9000 scanner (Fujifilm, Tokyo, Japan). All experiments were performed at least three times.

### Determination of cell viability

Cell viability was determined by MTT assay. Stably shSPTAN1-transfected cells were seeded in 96-well culture plates and incubated for 24 and 48 h, respectively. Culture medium was removed and replaced by 100 μl medium per well, containing MTT (Sigma, Munich, Germany) at a concentration of 833 μg/ml. Cells were then incubated for 2 h at 37°C, medium was removed and cells were air-dried. Finally, 100 μl decolorizing solution (DMSO containing 0.6% acetic acid and 0.1 g/ml SDS) was used to extract the purple dye from the cells. After 20 min, the absorbance of the colored solution was quantified at 570 nm in an Envision ELISA reader (PerkinElmer, Waltham, USA). The number of seeded cells was adjusted to 1 x 10^4^ cells per well, resulting in an absorbance lower than 1 for the untreated control, thus ensuring that measurements would remain in the linear range. Three independent experiments were carried out, each consisting of six replicates.

### Scratch wound migration assay

To analyze cell migration, the scratch wound migration assay and IncuCyte Zoom live cell imaging system (Essen Bioscience, Michigan, USA) were used. Cells were seeded into 96-well ImageLock plates (Essen Bioscience) and grown to confluence under standard conditions. As Caco-2 cells only loosely adhered to the well surface, ImageLock plates were pretreated with 5 μg collagen-1 (Corning, NY, USA) per well for 1 h at room temperature and washed with PBS before seeding. After 24 h, scratches were created simultaneously in all wells with a WoundMaker (Essen Bioscience) according to the manufacturer’s instructions. Wound closure was scanned every 4 h and monitored over 16 h using the Wound Width Analysis IncuCyte Software, allowing the exact identification of wound regions. The distance of cell migration in μm was calculated by subtracting the detected wound width from the initial wound width and dividing by 2. The experiment was performed in triplicate.

### Analysis of cell-cell contact

Cell-cell interactions were analyzed by transepithelial electrical resistance (TEER) measurements of stably transfected Caco-2 cell monolayers. Millicell polycarbonate transwell inserts were placed in 12-well culture plates, and each insert was lined with 1 x 10^5^ Caco-2 cells and cultured at 37°C and 5% CO_2_. Resistance measurements were carried out on alternate days using the Millicell ERS-2 Voltohmmeter. Resistivity was calculated for each well by subtracting background transepithelial resistance from the recorded resistance value, and then multiplying by the surface area of the membrane. Measurements were repeated on alternate days for a total of 30 days, and three independent experiments were carried out in duplicate.

### Determination of Caco-2 monolayer formation

Immunofluorescent staining techniques were used to confirm the presence of tight junctions in cell monolayers. Cells were incubated with 2% paraformaldehyde for 15 min at room temperature, then rendered permeable by exposure to 0.25% Triton X-100 in PBS for 15 min. Monolayers were incubated overnight at 4°C with primary rabbit antibody targeting the tight junction-associated protein zonula occludens-1 (ZO-1), diluted to 1:100 in PBS. A fluorescent-labeled goat anti-rabbit secondary antibody was then applied for 60 min at room temperature, diluted to 1:200 in PBS. Nuclei were counterstained by incubation with the intercalating agent DAPI for 30 min at room temperature, diluted to 1:5000 in distilled water. Transwell inserts were thoroughly washed with PBS between steps. Filter membranes were then excised using a scalpel and mounted onto slides for imaging using a Zeiss LSM 800 confocal scanning microscope.

### Immunohistochemical analysis

MLH1 and SPTAN1 expression was analyzed by immunohistochemistry using paraffin-embedded, invasively growing MLH1-deficient or MLH1-proficient colorectal tumor tissue, as described previously [15].

Briefly, 2 μm sections of representative samples were cut from paraffin-embedded, invasively growing colorectal carcinoma specimens. Surrounding normal colonic mucosa served as an internal control. Sections were deparaffinized twice with xylene and rehydrated in five graded alcohol baths. Antigen retrieval by heating was performed in a pressure cooker for 15 min in EDTA buffer, pH 8.0. This was followed by incubation for 10 min with 3% H_2_O_2_ to block endogenous peroxidase activity. Sections were washed with 1x PBS (Gibco, USA) before and in between incubation steps. Primary MLH1 antibody (clone G168-728, 1:500 dilution) or primary SPTAN1 antibody (clone C-11, 1:250 dilution) were diluted in PBS containing 1% BSA. Sections were incubated with one primary antibody at 4°C overnight, followed by application of the EnVision System mouse (K4000, Agilent, USA), which employs the enzyme horseradish peroxidase and the chromogen 3,3’-diaminobenzidine (DAB). Samples were treated with the peroxidase reagent DAB for 10 min, diluted to 1 drop of DAB chromogen per ml of DAB substrate buffer (K3467, Agilent, USA). Sections were counterstained using Gill’s hematoxylin solution. Immunohistochemical staining was examined using a Keyence microscope (Model BZ-9000, KEYENCE Co., Osaka, Japan). Negative controls were processed in parallel to exclude non-specific staining.

### Image acquisition and processing

Representative images for detection of MLH1 and SPTAN1 were captured at 10-fold magnification using a Keyence BZ-9000 optical microscope. MLH1 status was determined for each tumor based on the presence or absence of enterocyte nuclear staining, as confirmed by a consultant pathologist. SPTAN1 levels were quantified using a simple computer-based algorithm in ImageJ. First, images were cropped to separate normal mucosa from malignant tissue, and to exclude non-parenchymal tissue from the analysis. Files were then converted to 8-bit grayscale, in order to assign to each pixel a value between zero and 255. Consequently, intensely stained pixels were assigned high numerical intensity values, whereas low intensity values were allocated to unstained low-intensity background pixels. Finally, a mean intensity value for all pixels in a given image was computed by the software, applying a threshold value of 70 to exclude from the analysis non-specific background intensity, as well as tissue- or parenchyma-free areas with low-intensity background pixels.

### Analysis of MSI and the *BRAF* V600E mutation

All MLH1-deficient tumor samples were additionally tested for MSI and the *BRAF* V600E mutation. MSI analysis and determination of the *BRAF* V600E mutation were performed using the Idylla platform, a fully automated, real-time PCR-based molecular diagnostics system (Biocartis NV, Mechelen, Belgium). Idylla analyses were carried out according to the manufacturer’s recommendations regarding the minimum tissue size, tissue quality, and tumor content required for a valid workflow. It is recommended that a tissue area in the range of 25–300 mm^2^, containing at least 50% tumor cells, be analyzed. In brief, 2 x 5 μm of macro-dissected, paraffin-embedded tumor sections were prepared on microscope slides, spread between two wetted (nuclease-free water) filter papers, placed into the so-called Idylla cartridges and introduced into the instrument. Paraffin and tissue disruption, DNA extraction, as well as special PCRs were then automatically performed. Idylla MSI cartridges contain and analyze a set of seven MSI Biomarkers, consisting of short quasi-monomorphic mononucleotide repeats located in the *ACVR2A*, *BTBD7*, *DIDO1*, *MRE11*, *RYR3*, *SEC31A* and *SULF2* genes [24].

Idylla BRAF cartridges are capable of detecting the *BRAF* wild type and the following mutations: *BRAF* V600E, *BRAF* V600E2, *BRAF* V600D, *BRAF* V600K, *BRAF* V600R and *BRAF* V600M [25, 26]. *BRAF* V600E, V600E2 and V600D mutations are detected as the “V600E/E2/D mutation”, and V600K, V600R and V600M mutations as the “V600K/R/M mutation” by the system.

### Statistical analysis

Data are expressed as means ± SEM as appropriate. Tests for comparison of quantitative markers were selected after testing for normal distribution using the Kolmogoroff-Smirnov-Lilliefors test. Immunohistochemistry data was assessed for statistical significance between groups by Student’s t-test and Fisher’s exact test. For comparison of MTT assays, the Kruskal-Wallis test followed by *post hoc* tests with Bonferroni-Holm correction was applied. Differences between mean resistivity values (TEER measurements) were assessed for statistical significance using one-way ANOVA and *post hoc* Scheffe analysis.

*P* values are two-sided and values <0.05 are considered statistically significant. Data were analyzed using the software BiAS for Windows (version 9.11) [27].

## Results

### Strong expression of SPTAN1 correlates with MLH1 proficiency in colon tumors

**Fig 1:**
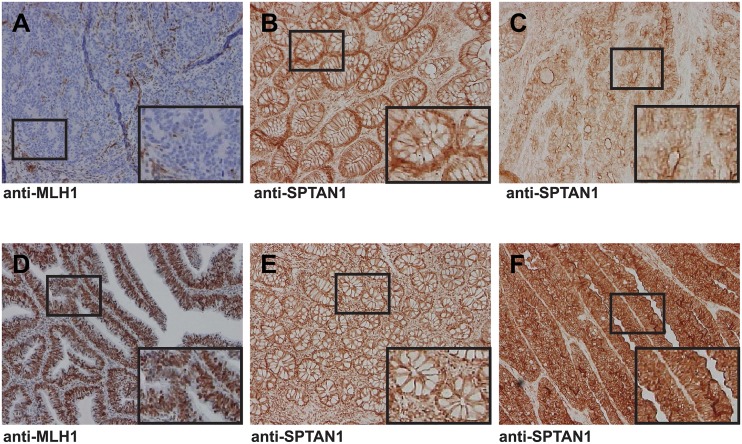
Analysis of SPTAN1 expression in MLH1-deficient and MLH1-proficient CRCs. Sample immunohistochemistry images of an MLH1-deficient CRC show (A) loss of MLH1, (B) moderate SPTAN1 expression in the adjacent normal mucosa, and (C) a clear reduction of SPTAN1 expression in the tumor. The sporadic CRC below shows (D) intense nuclear staining for MLH1, (E) moderate SPTAN1 expression in the adjacent normal mucosa, and (F) strong SPTAN1 expression in the tumor. Note that SPTAN1 is located basolaterally in the normal mucosa, apically in MLH1-deficient tumor tissue, and is distributed across the cytoplasm in MLH1-proficient tumor tissue. Original images were captured at 10-fold magnification, and the area outlined by a rectangle was magnified 40-fold for better visualization.

First, CRC tissue and surrounding normal mucosa from 189 patients was analyzed for MLH1 expression by immunohistochemistry (sample images shown in Fig 1A+1D). 162 CRCs were MLH1-proficient (86%), whereas 27 CRCs showed lack of MLH1 expression and were classified as MLH1-deficient (14%) (Table 1, S1 Table). In order to gain insights into the role of SPTAN1 in CRC progression, we then used immunohistochemistry to determine the expression of SPTAN1 in tumor tissue (sample images shown in Fig 1C+1F) and (if available) in the corresponding surrounding normal mucosa (sample images shown in Fig 1B+1E), and quantified values using a simple computer-based algorithm in ImageJ (see Materials and methods). The immunohistochemical staining of controls which were processed in parallel was negative (S1 Fig).

Next, the SPTAN1 intensities of tumor tissue and normal mucosa were compared. In total, normal mucosal tissue was available for 152 of the 189 patient samples. Quantitation of SPTAN1 was successful in 98.4% of all cases. Three cases required readjustments due to high background signals (1.6%) (S1 Table).

**Fig 2:**
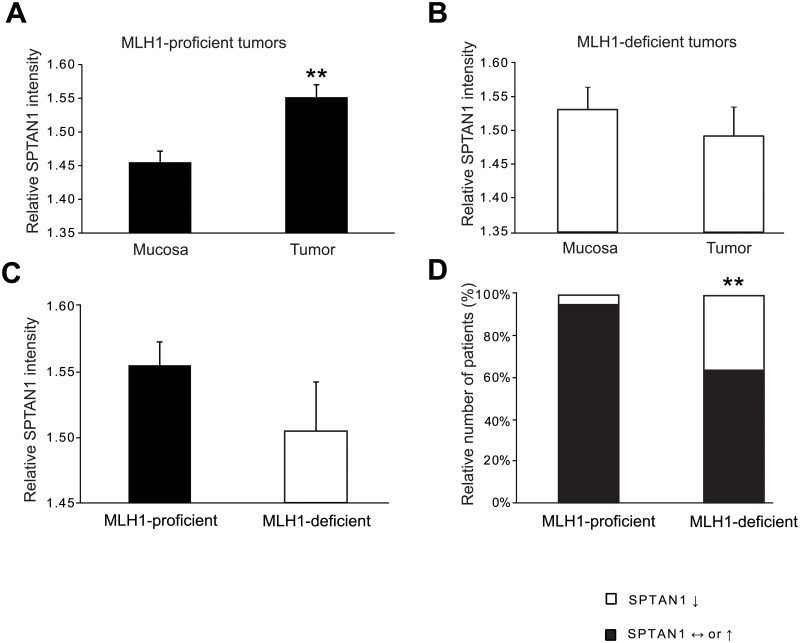
Comparison of SPTAN1 in MLH1-proficient vs. MLH1-deficient CRCs. The intensity of protein expression was determined by immunohistochemistry to compare SPTAN1 expression in CRC tissue from 189 patients. SPTAN1 expression of (A) MLH1-proficient tumors and (B) MLH1-deficient tumors was compared with the surrounding mucosal tissue. (C) SPTAN1 expression of MLH1-deficient vs. MLH1-proficient tumors was analyzed. (D) The general dependence between MLH1 status and SPTAN1 intensity was evaluated. SPTAN1 was significantly increased in MLH1-proficient tumors compared with the normal mucosa, while MLH1-deficient tumors showed a clear trend of decreased SPTAN1 levels compared with surrounding normal tissue. MLH1-proficient tumors showed non-significantly enhanced SPTAN1 levels compared with MLH1-deficient tumors, and a strong correlation between MLH1 status and SPTAN1 intensity was detected. Data are expressed as means ± SEM as appropriate. Statistical significance was assessed by Student’s t-test and Fisher’s exact test. *P* values are two-sided and values <0.05 (*) or <0.005 (**) are considered statistically significant.

As shown in Fig 2A, we detected significantly increased SPTAN1 levels in MLH1-proficient tumor tissue compared with normal mucosa (p<0.0001). In contrast, MLH1-deficient tumors tended towards lower SPTAN1 intensities compared with the corresponding mucosal tissue (p = 0.462) (Fig 2B).

Comparing the SPTAN1 expression of all tumor tissues, we found a trend of reduced SPTAN1 expression in MLH1-deficient vs. MLH1-proficient tumors (p = 0.271) (Fig 2C).

In addition, the general dependence between MLH1 status and SPTAN1 intensity was evaluated. Overall, 96.1% of MLH1-proficient tumors and 62.5% of MLH1-deficient tumors showed as much or increased SPTAN1 intensity compared with normal mucosa, whereas SPTAN1 expression was reduced in 37.5% of MLH1-deficient but only 3.9% of MLH1-proficient tumors (Fig 2D, S2 Table). Altogether, we identified a strong correlation between MLH1 status and SPTAN1 intensity (p<0.0001).

### Stage II to IV and metastatic CRCs are associated with low SPTAN1 levels

SPTAN1 is a cytoskeletal protein which is involved in cell adhesion and intercellular communication [20], and the cellular amount of SPTAN1 might have a significant impact on the tumor progression of CRCs. In order to identify a potential connection between levels of SPTAN1, tumor stage and metastasis of CRCs, we separated our cohort into stages I, II, III and IV CRCs and determined the corresponding SPTAN1 levels. Furthermore, we grouped CRCs into metastatic and non-metastatic tumors and analyzed their respective SPTAN1 expression levels.

**Fig 3:**
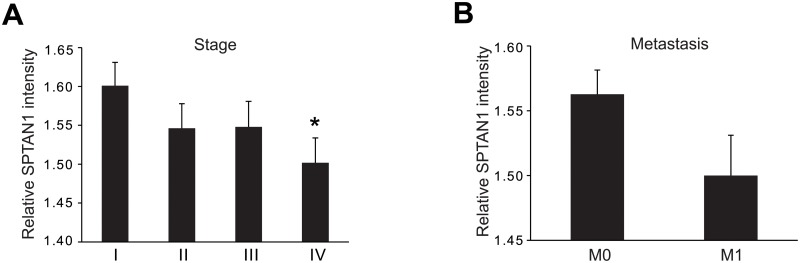
Low levels of SPTAN1 correlate with advanced tumor stage and metastasis. To correlate tumor stage or metastasis and SPTAN1 expression levels, our cohort of CRCs were separated into stages I, II, III and IV, and the corresponding SPTAN1 levels were determined in these groups. (A) Stage I CRCs correlated with higher relative SPTAN1 intensity, while stages II to IV showed a gradual reduction of SPTAN1, and SPTAN1 expression was significantly reduced in stage IV compared with stage I CRCs. (B) Relative SPTAN1 intensity was lower in metastatic compared with non-metastatic CRCs. Data are expressed as means ± SEM as appropriate. Statistical significance was assessed by Student’s t-test. *P* values are two-sided and values <0.05 (*) are considered statistically significant.

As shown in Fig 3A, we found that stage I CRCs correlated with significantly higher SPTAN1 levels compared with stage IV CRCs (p = 0.027). Stages II to IV showed a gradual reduction of SPTAN1 expression (Fig 3A). Moreover, when we compared metastatic CRCs with non-metastatic CRCs, we detected much lower levels of SPTAN1 in metastatic CRCs (Fig 3B). This correlation could be determined in MMR-proficient but also in the group of MLH1-deficient CRCs (data not shown).

### Reduction of SPTAN1 partly impairs cell proliferation

**Fig 4:**
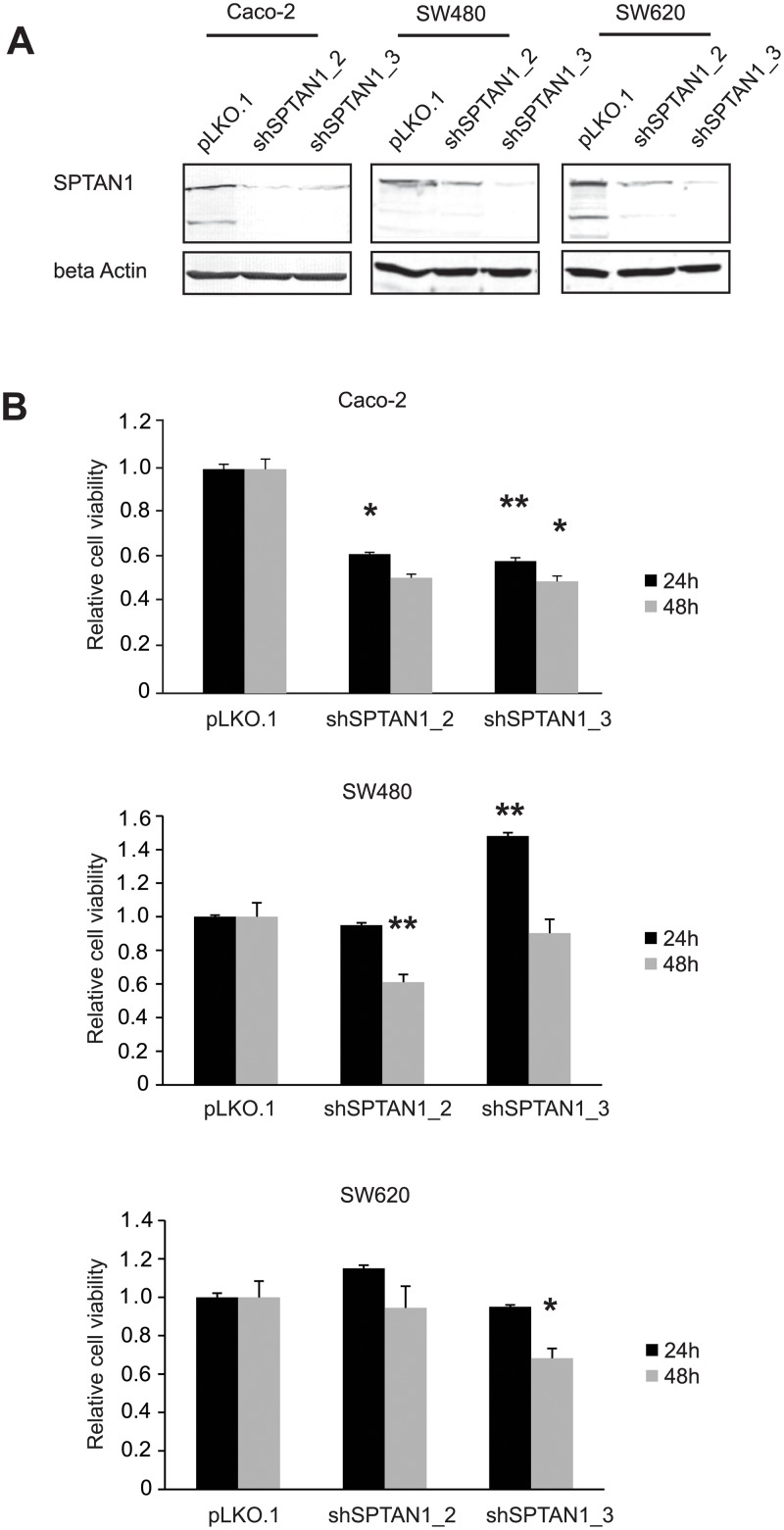
SPTAN1 knockdown cells show decreased cell proliferation. Two different Caco-2, SW480 and SW620 SPTAN1 knockdown cell lines were generated using lentiviral transduction, and (A) the success of SPTAN1 knockdown was verified by Western blotting. (B) Relative cell viability of shSPTAN1_2/shSPTAN1_3-transfected Caco-2, SW480 and SW620 cells was analyzed by MTT assay after 24 h and 48 h, in parallel with the mock-transfected controls. Comparison of relative cell viability was performed by setting the pLKO.1-transfected controls to 1. The shSPTAN1_2 knockdown led to a significant reduction of Caco-2 cell viability after 24 h, and of SW480 cells after 48 h. The shSPTAN1_3 knockdown induced significantly decreased viability after both 24 and 48 h in Caco-2 cells, and after 48 h in SW620 cells. Data are expressed as means ± SEM as appropriate. For comparison of MTT assays, the Kruskal-Wallis test followed by a *post hoc* Bonferroni-Holm correction was applied. *P* values are two-sided and values <0.05 (*) or <0.005 (**) are considered statistically significant.

Stably shSPTAN1-transfected Caco-2, SW480 and SW620 cell lines were generated using lentiviral transduction, and success of SPTAN1 knockdown was verified by Western blotting using the anti-SPTAN1 antibody MAB1622 (Fig 4A, corresponding whole Western blots depicted in the S2 Fig). The result was confirmed using the anti-SPTAN1 antibody C-11, which was normally used for immunochemical staining (S3 Fig).

Cell viability of Caco-2, SW480 and SW620 cells with decreased SPTAN1 expression was determined by MTT assay 24 and 48 h after incubation, and compared with the control cell line. Reduction of SPTAN1 led to significantly impaired cell viability after 24 h (p = 0.027), and a clear trend after 48 h (p = 0.104) in shSPTAN1_2-transfected Caco-2 cells (Fig 4B, upper panel). shSPTAN1_3-transfected Caco-2 cells showed significantly reduced cell viability both 24 and 48 h after incubation (p = 0.002 and p = 0.027, respectively) (Fig 4B, upper panel). The knockdown of SPTAN1 in shSPTAN1_2-transfected SW480 cells led to significantly impaired cell viability after 48 h (p = 0.000), while shSPTAN1_3-transfected SW480 showed higher cell viability than the control cell line after 24 h (p = 0.000) and no difference after 48 h (Fig 4B, middle panel). shSPTAN1_3-transfected SW620 cells showed a significant decrease in cell viability after 48 h (p = 0.013, Fig 4B, lower panel), while the knockdown using shSPTAN1_2 showed no difference (Fig 4B, lower panel).

### SPTAN1 knockdown reduces cell mobility

**Fig 5:**
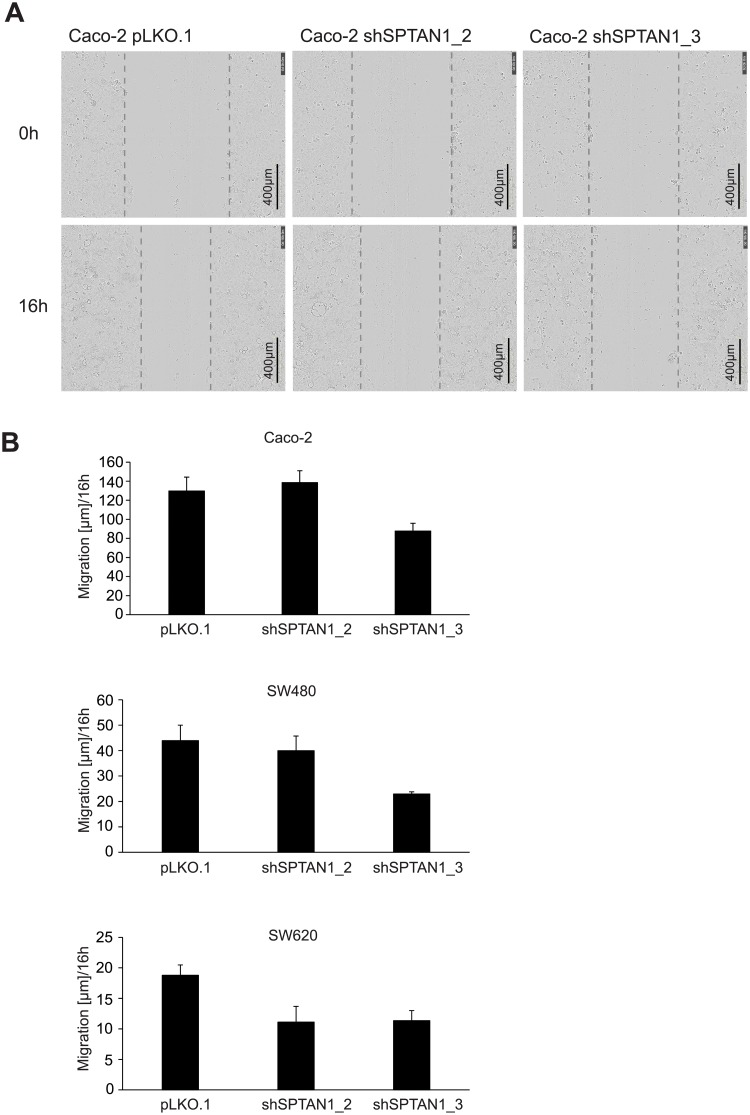
Decreased SPTAN1 expression leads to reduced cell migration. The wound migration assay and IncuCyte Zoom live cell imaging system were used to test the influence of SPTAN1 reduction on cell mobility. The migratory rates of shSPTAN1-transfected Caco-2, SW480, SW620 and control-transfected sister cells were compared by measuring the alteration of the wound width at different time points (0 h, 3 h, 6 h, 12 h and 16 h). (A) Representative corresponding images were taken of Caco-2 cell lines immediately after scratching the cultures (0 h) and 16 h later, at 400-fold magnification. (B) The shSPTAN1_3 knockdown induced impaired cell mobility in Caco-2, SW480 as well as SW620 cells, and the shSPTAN1_2 knockdown reduced cell mobility in SW620 cells. Graphs indicate the results (mean ± S.D.) of at least three independent experiments.

Cell mobility was analyzed using the scratch wound migration assay and IncuCyte Zoom live cell imaging system. To test the influence of SPTAN1 reduction on cell mobility, the migratory rate of shSPTAN1-transfected Caco-2, SW480, SW620 and control-transfected sister cells was compared in a migration assay by measuring the alteration of the wound width at different time points (0 h, 3 h, 6 h, 12 h and 16 h). The results at 0 h and 16 h are shown in Fig 5A+5B. The mobility of shSPTAN1_3-transfected Caco-2, SW480 and SW620 cells was decreased. In terms of shSPTAN1_2-transfected cells, only the SW620 cell line showed a clear decrease in cell mobility.

### Loss of SPTAN1 expression reduces the ability for cell-cell contact

**Fig 6:**
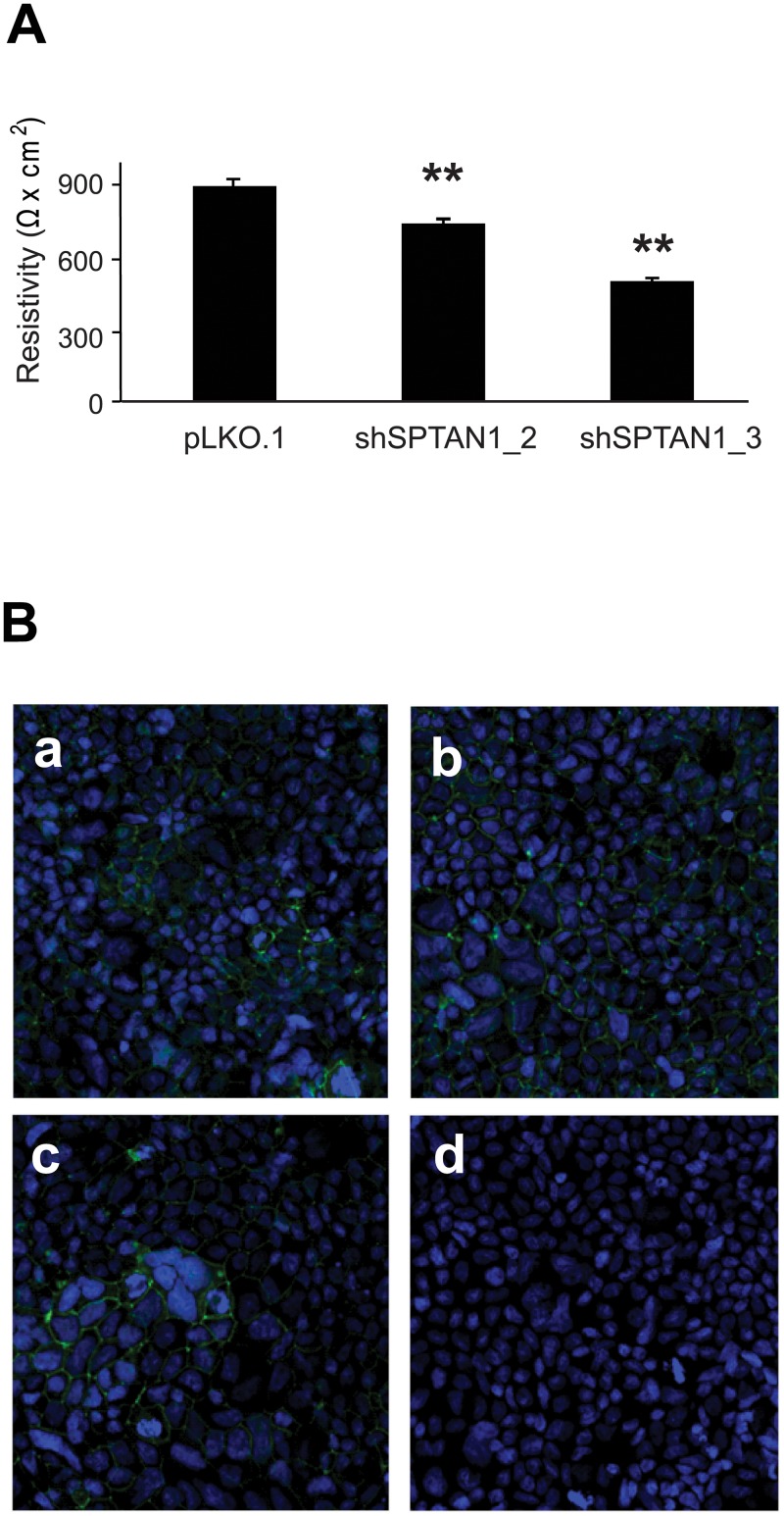
SPTAN1 reduction impairs cell-cell contact of Caco-2 cells. To determine the influence of SPTAN1 on cell-cell contact, shSPTAN1-transfected Caco-2 cells were analyzed by TEER measurements. (A) Monolayer resistivity for each cell line was measured after 20 days and compared with pLKO.1-transfected controls. SPTAN1 knockdown resulted in a significant reduction in monolayer resistivity values. Data are expressed as means ± SEM as appropriate. Differences between mean resistivity values were assessed for statistical significance using one-way ANOVA and *post hoc* Scheffe analysis. *P* values are two-sided and values <0.005 (**) are considered statistically significant. (B) Monolayer formation of Caco-2 cells transduced with (a) pLKO.1, (b) shSPTAN1_2 or (c) shSPTAN1_3 was verified after day 20. Cells were fixed with paraformaldehyde, rendered permeable by exposure to Triton X-100, incubated with anti-ZO-1 antibody, and subsequently with fluorescent-labeled goat anti-rabbit secondary antibody. Nuclei were counterstained with DAPI. Finally, filter membranes were excised and mounted onto slides for imaging using a Zeiss LSM 800 confocal scanning microscope. (d) Negative control cells omitting the primary antibody were processed in parallel. There was no detectable phenotypic difference between monolayers from different cell lines.

TEER measurements were performed to analyze the influence of reduced SPTAN1 expression on cell-cell contact. As shown in Fig 6A, decreased SPTAN1 expression led to significantly lower monolayer resistivity values for Caco-2 cells transfected with shSPTAN1_2 (p = 0.001) or shSPTAN1_3 (p = 0.001), compared with the pLKO.1-transfected control. The formation of monolayers was verified by immunofluorescent staining with anti-ZO-1 antibodies, analyzed using a confocal scanning microscope (sample images shown in Fig 6B).

## Discussion

In the current study, we used paraffin-embedded tumors and adjacent normal mucosa from 189 patients to investigate the correlation of MLH1 and SPTAN1 expression in CRCs. We found evidence for a close correlation between MLH1 and SPTAN1 expression levels, and between the amount of SPTAN1 and tumor progression and metastasis.

To overcome the limitations of laborious manual semi-quantitative scoring of immunohistochemically stained tissue sections, more objective, reproducible, and rapid computer-assisted quantification methods have been developed and applied to digital images of tissue sections [28, 29]. We quantified immunohistochemically detected SPTAN1 expression using ImageJ. Images of the slides were obtained using a Keyence BZ-9000 optical microscope, and SPTAN1 levels were evaluated by converting color images to grayscale and applying a simple computer-based algorithm. Using this method, successful quantification of SPTAN1 was possible in 98.4% of all paraffin-embedded samples. The algorithm can therefore be considered a reliable method for analysis of SPTAN1 intensity.

Looking at the SPTAN1 results in detail, we found a clear correlation between MLH1 deficiency and SPTAN1 reduction. Around 40% of the MLH1-deficient tumors showed significantly decreased SPTAN1 expression. These results are in line with previously published data [15]. Using different cell lines and a small number of patient samples, Hinrichsen *et al*. demonstrated that loss of MLH1 is associated with impaired SPTAN1 expression [15]. However, around 60% of the tested MLH1-deficient tumors from our cohort showed as much or increased SPTAN1 intensity compared with the surrounding normal mucosa. A possible explanation for the divergent SPTAN1 levels within the MLH1-deficient tumor group could be that the influence of MLH1 on SPTAN1 expression is indirect and occurs at the mRNA level [15]. One might postulate that an increased mutation rate at microsatellite loci, caused by MMR deficiency, differentially affects specific SPTAN1-associated transcription factors. Modulation of transcription factor binding by microsatellite length changes has been demonstrated for several genes [30]. Some of the earliest evidence was reported in 2001, for a (TACT)5–10 repeat in the first intron of the human TH gene [31]. This microsatellite binds the transcription factor HBP1 and the zinc finger protein ZNF191, and exerts a copy number-dependent silencing effect on the gene [31]. Depending on the type of mutation, the postulated SPTAN1-correlated transcription factors might induce 1) downregulation of SPTAN1 in those tumors which show decreased SPTAN1 expression or 2) upregulation of SPTAN1 in those tumors which show increased SPTAN1 intensity, and 3) no effect on SPTAN1 expression in MLH1-deficient tumors that express as much SPTAN1 as the normal mucosa. However, this hypothesis has to be explored in detail by further experiments. Furthermore, SPTAN1 expression might be affected at the protein level by stabilization or degradation of SPTAN1, as shown by Lefferts *et al*. [32] and others who identified cleaved breakdown products of SPTAN1 during apoptosis [21, 33].

Next, we analyzed the importance of the expression level of SPTAN1 for CRC progression. We found that early stage I CRCs correlated with higher SPTAN1 levels than stages II to IV, which showed a gradual reduction of SPTAN1 levels. In addition, SPTAN1 levels were lower in metastatic compared with non-metastatic tumors. Tumor progression and metastasis is characterized by striking morphological changes which enable cancer cell movement and depend on pronounced switches of the expression levels of genes involved in cell adhesion and attachment [34, 35]. We therefore surmised that the expression of the cytoskeletal protein SPTAN1 might be critical for cell growth, migration and attachment. To test this hypothesis, cell viability and motility was analyzed using two stably shSPTAN1-transfected Caco-2, SW480 and SW620 cell lines, respectively. In addition, cell-cell contact measurements were performed with both SPTAN1 knockdown Caco-2 strains (since neither SW480 nor SW620 cells formed monolayers). A decrease of SPTAN1 expression induced impaired cellular viability and mobility in all three tested cancer cell lines, underlining the relevance of SPTAN1 for cell growth and migration. Moreover, monolayers of Caco-2 cells deficient in SPTAN1 exhibited reduced resistivity values compared with controls, and the degree of TEER reduction was dependent on SPTAN1 knockdown efficiency. Therefore, the amount of SPTAN1 appears to be essential for the strength of cell-cell interactions. Fittingly, the involvement of SPTAN1 in adhesion processes and establishing cell-cell contact has been described by several groups [20, 36–39]. In the context of tumor aggressiveness, we speculate that high SPTAN1 expression counteracts metastasis by ensuring epithelial cohesion and tight junctional integrity. The adhesive power imparted by SPTAN1 may promote the formation of strong cell-cell contacts within the primary tumor, thus preventing extravasation and the formation of distant metastases [35, 40]. However, it remains to be discussed how the reduced expression of SPTAN1 in MLH1-deficient tumors can be explained, as these tumors are described to have a better prognosis than sporadic CRCs [10]. SPTAN1 expression might decrease along with the loss of epithelial polarity accompanying tumor development and progression [41, 42], which would actually mean that lower SPTAN1 expression favors the metastatic ability of tumor cells. Yet, it is conceivable that this may not have negative effects in patients with MSI-H CRCs. In the majority of patients with MSI-H tumors it has been shown that the immune system is able to recognize tumor cells [43]. Circulating tumor cells that have separated from the primary tumor might be eliminated more successfully in these patients, before they can form metastases. However, this hypothesis has to be confirmed by further investigations.

In summary, we demonstrated that loss of MLH1 is correlated with reduced expression of SPTAN1. In addition, we showed that SPTAN1 has impact on cell viability, mobility and cell-cell contact, and that its expression level correlates with cancer progression. We therefore recommend the determination of SPTAN1 levels in CRCs as a useful marker to predict cancer aggressiveness.

## Supporting information

S1 Fig Negative controls of immunohistochemical staining omitting the primary antibody, to exclude non-specific staining of (A) MLH1 and (B) SPTAN1 antibodies. Sections were processed in parallel with normal staining.
(EPS)

S2 Fig Whole Western blots corresponding to the cropped blots shown in Fig 4A. SPTAN1, MLH1 and Actin beta expression levels of (A) Caco-2 SPTAN1 knockdown cells, and (B) SW480 and SW620 SPTAN1 knockdown cells. Control cells were transduced with the same amount of viral vector containing non-mammalian shRNA (pLKO.1).
(EPS)

S3 Fig The success of shSPTAN1 knockdown, determined in Fig 4A using the anti-SPTAN1 antibody MAB1622, was confirmed using the anti-SPTAN1 antibody C-11. The antibody was diluted to 1:100 in TBS-T with 5% milk, and incubated at 4°C overnight. SPTAN1 and beta Actin expression levels of Caco-2, SW480 and SW620 SPTAN1 knockdown cells as well as controls are shown. Control cells were transduced with the same amount of viral vector containing non-mammalian shRNA (pLKO.1).
(EPS)

S1 Table Clinicopathological characteristics of 189 patients with colorectal cancer evaluated for MLH1 and SPTAN1 expression. (DOCX)

S2 Table Comparison of SPTAN1 expression. (DOCX)

## Abbreviations

CRC: Colorectal cancer
DAB: 3,3’-diaminobenzidine
MLH1: MutL homolog 1
MMR: DNA mismatch repair
MSH2: MutS homolog 2
MSI-H: microsatellite instability-high
TEER: transepithelial electrical resistance
SPTAN1: non-erythroid spectrin αII
ZO-1: Zonula occludens-1
