# Supplementary material for: Downregulation of SPTAN1 is related to MLH1 deficiency and metastasis in colorectal cancer
Source: PLoS One. 2019 Mar 11;14(3):e0213411. doi: 10.1371/journal.pone.0213411 (PMC6411122; doi:10.1371/journal.pone.0213411)
Supplement: S1 Table — (DOCX) [file pone.0213411.s004.docx]

**S1 Table. Clinicopathological characteristics of 189 patients with colorectal cancer evaluated for MLH1 and SPTAN1 expression**

| **Patient** | **Gender^1)^** | **Localization relative to the splenic flexure** | **Age at diagnosis** | **Year of diagnosis and surgery** | **Tumor** | **Metastases^2)^** | **Stage** | **MLH1 status** | **Mucosa**  **(SPTAN1 intensity)** | **Tumor**  **(SPTAN1 intensity)** | **Change (%)** | **Compared to Mucosa** | **MSI-H** | **BRAF wt /**  **BRAF V600^E/E2/D^** |
| --- | --- | --- | --- | --- | --- | --- | --- | --- | --- | --- | --- | --- | --- | --- |
| 1 | F | Distal | 34 | 2011 | pT1 | M0 | I | + | 1,53 | 1,56 | 2 | >^*^ |  |  |
| 2 | F | Proximal | 62 | 2014 | pT2 | M0 | I | + | 1,65 | 1,83 | 11 | > |  |  |
| 3 | M | Proximal | 77 | 2012 | pT1 | M0 | I | + | 1,41 | 1,56 | 11 | > |  |  |
| 4 | M | Distal | 84 | 2015 | pT3 | M0 | IIA | + | 0,00 | 1,81 |  |  |  |  |
| 5 | M | Proximal | 75 | 2014 | pT4b | M0 | IIC | + | 1,78 | 1,61 | -10 | < |  |  |
| 6 | F | Distal | 74 | 2014 | pT3 | M0 | IIIB | + | 0,00 | 1,67 |  |  |  |  |
| 7 | F | Proximal | 69 | 2015 | pT3 | M0 | IIIB | + | 0,00 | 1,97 |  |  |  |  |
| 8 | M |  | 79 | 2012 | pT2 | M1a (HEP) | IVA | + | 1,25 | 1,29 | 4 | = |  |  |
| 9 | F | Proximal | 70 | 2013 | pT3 | M1 (HEP) | IV | + | 1,39 | 1,50 | 8 | = |  |  |
| 10 | M | Proximal | 77 | 2014 | pT1(sm) | M0 | I | + | 0,00 | 1,65 |  |  |  |  |
| 11 | F | Distal | 49 | 2014 | pT1(sm3) | M0 | I | + | 1,25 | 1,54 | 23 | > |  |  |
| 12 | F | Proximal | 69 | 2011 | pT1a | M0 | I | + | 1,68 | 1,84 | 9 | = |  |  |
| 13 | M | Distal | 73 | 2013 | pT1(sm2) | M0 | I | + | 1,28 | 1,38 | 7 | = |  |  |
| 14 | M | Proximal | 88 | 2013 | pT2 | M0 | I | + | 0,00 | 1,45 |  |  |  |  |
| 15 | F | Distal | 58 | 2013 | pT2 | M0 | I | + | 1,23 | 1,48 | 20 | > |  |  |
| 16 | F | Distal | 57 | 2013 | pT1 | M0 | I | + | 1,38 | 1,41 | 2 | = |  |  |
| 17 | F | Proximal | 74 | 2015 | pT2 | M0 | I | + | 0,00 | 1,84 |  |  |  |  |
| 18 | M | Distal | 75 | 2012 | pT2 | M0 | I | + | 1,39 | 1,54 | 11 | > |  |  |
| 19 | M | Proximal | 70 | 2012 | pT2 | M0 | I | + | 1,54 | 1,42 | -8 | = |  |  |
| 20 | F | Proximal | 65 | 2013 | pT1(sm2) | M0 | I | + | 1,64 | 1,82 | 11 | > |  |  |
| 21 | M | Distal | 60 | 2015 | pT2 | M0 | I | + | 1,86 | 1,84 | -1 | >^*^ |  |  |
| 22 | M | Distal | 52 | 2014 | pT2 | M0 | I | + | 0,00 | 1,50 |  |  |  |  |
| 23 | F | Proximal | 61 | 2014 | pT2 | M0 | I | + | 1,50 | 1,64 | 9 | = |  |  |
| 24 | M | Proximal | 65 | 2013 | pT2 | M0 | I | + | 1,42 | 1,56 | 10 | > |  |  |
| 25 | M | Proximal | 78 | 2014 | pT2 | M0 | I | + | 1,55 | 1,73 | 12 | > |  |  |
| 26 | M | Proximal | 72 | 2015 | pT2 | M0 | I | + | 1,66 | 1,86 | 12 | > |  |  |
| 27 | M | Distal | 76 | 2014 | pT2 | M0 | I | + | 1,30 | 1,72 | 32 | > |  |  |
| 28 | M | Proximal | 84 | 2012 | ypT2 | M0 | I | + | 1,25 | 1,36 | 9 | = |  |  |
| 29 | M | Proximal | 60 | 2011 | pT1 | M0 | I | + | 1,57 | 1,73 | 10 | > |  |  |
| 30 | F | Distal | 59 | 2012 | pT2 | M0 | I | + | 1,12 | 1,12 | 0 | = |  |  |
| 31 | M | Proximal | 73 | 2013 | pT2 | M0 | I | + | 0,00 | 1,50 |  |  |  |  |
| 32 | M | Distal | 91 | 2014 | pT2 | M0 | I | + | 1,65 | 1,89 | 14 | > |  |  |
| 33 | M | Proximal | 60 | 2013 | pT3 | M0 | IIA | + | 1,56 | 1,26 | -19 | < |  |  |
| 34 | M | Distal | 76 | 2013 | pT3 | M0 | IIA | + | 0,00 | 1,34 |  |  |  |  |
| 35 | M | Distal | 78 | 2015 | pT3 | M0 | IIA | + | 0,00 | 2,04 |  |  |  |  |
| 36 | M | Proximal | 78 | 2016 | pT3 | M0 | IIA | + | 2,16 | 2,07 | -4 | = |  |  |
| 37 | F | Proximal | 79 | 2014 | pT3 | M0 | IIA | + | 0,00 | 1,81 |  |  |  |  |
| 38 | M | Distal | 60 | 2012 | pT3 | M0 | IIA | + | 1,61 | 1,80 | 12 | > |  |  |
| 39 | F | Proximal | 85 | 2013 | pT3 | M0 | IIA | + | 1,20 | 1,36 | 14 | > |  |  |
| 40 | M | Proximal | 80 | 2013 | pT3 | M0 | IIA | + | 0,00 | 1,32 |  |  |  |  |
| 41 | M | Proximal | 87 | 2014 | pT3 | M0 | IIA | + | 1,59 | 1,90 | 19 | > |  |  |
| 42 | F | Proximal | 70 | 2015 | pT3 | M0 | IIA | + | 0,00 | 2,06 |  |  |  |  |
| 43 | F | Distal | 74 | 2012 | pT3m | M0 | IIA | + | 1,77 | 1,54 | -13 | < |  |  |
| 44 | M | Proximal | 74 | 2011 | pT3 | M0 | IIA | + | 0,00 | 1,31 |  |  |  |  |
| 45 | M | Proximal | 76 | 2013 | pT3 | M0 | IIA | + | 0,00 | 1,32 |  |  |  |  |
| 46 | F | Proximal | 63 | 2016 | pT3 | M0 | IIA | + | 1,26 | 1,14 | -9 | = |  |  |
| 47 | M | Proximal | 51 | 2016 | pT3 | M0 | IIA | + | 1,40 | 1,38 | -2 | = |  |  |
| 48 | M | Distal | 52 | 2014 | pT3 | M0 | IIA | + | 1,45 | 1,46 | 1 | = |  |  |
| 49 | F | Distal | 73 | 2012 | pT3 | M0 | IIA | + | 1,51 | 1,59 | 6 | = |  |  |
| 50 | F | Proximal | 73 | 2012 | pT3 | M0 | IIA | + | 1,35 | 1,38 | 2 | = |  |  |
| 51 | M | Distal | 83 | 2014 | pT3 | M0 | IIA | + | 1,45 | 1,47 | 1 | = |  |  |
| 52 | M | Proximal | 69 | 2013 | pT3 | M0 | IIA | + | 1,39 | 1,46 | 6 | = |  |  |
| 53 | M | Proximal | 53 | 2012 | pT3 | M0 | IIA | + | 1,34 | 1,46 | 9 | = |  |  |
| 54 | F | Proximal | 75 | 2015 | pT3 | M0 | IIA | + | 1,88 | 1,97 | 5 | = |  |  |
| 55 | M | Proximal | 87 | 2012 | pT3 | M0 | IIA | + | 1,38 | 1,43 | 4 | = |  |  |
| 56 | F | Proximal | 83 | 2013 | pT3 | M0 | IIA | + | 1,53 | 1,60 | 4 | = |  |  |
| 57 | M | Proximal | 58 | 2011 | pT3 | M0 | IIA | + | 1,59 | 1,75 | 10 | > |  |  |
| 58 | M | Distal | 81 | 2013 | pT3 | M0 | IIA | + | 1,32 | 1,57 | 19 | > |  |  |
| 59 | F | Proximal | 77 | 2012 | pT3 | M0 | IIA | + | 0,00 | 1,19 |  |  |  |  |
| 60 | M | Distal | 72 | 2012 | pT3 | M0 | IIA | + | 0,00 | 1,36 |  |  |  |  |
| 61 | F | Proximal | 82 | 2012 | pT3 | M0 | IIA | + | 0,00 | 1,14 |  |  |  |  |
| 62 | F | Proximal | 62 | 2012 | pT3 | M1 (PUL) | IIA | + | 1,51 | 1,82 | 20 | > |  |  |
| 63 | M | Distal | 80 | 2012 | pT4a | M0 | IIB | + | 1,25 | 1,26 | 1 | = |  |  |
| 64 | M |  | 88 | 2015 | pT4a | M0 | IIB | + | 0,00 | 1,97 |  |  |  |  |
| 65 | M | Proximal | 78 | 2013 | pT4b | M0 | IIC | + | 1,24 | 1,43 | 15 | > |  |  |
| 66 | M | Distal | 81 | 2013 | pT4b | M0 | IIC | + | 1,21 | 1,61 | 33 | > |  |  |
| 67 | M | Distal | 75 | 2013 | pT4b | M0 | IIC | + | 1,44 | 1,32 | -8 | = |  |  |
| 68 | M | Distal | 65 | 2016 | pT4b | M0 | IIC | + | 0,00 | 1,72 |  |  |  |  |
| 69 | M | Proximal | 74 | 2011 | pT3 | M0 | III | + | 0,00 | 1,37 |  |  |  |  |
| 70 | M | Distal | 67 | 2016 | pT2 | M0 | IIIA | + | 1,24 | 1,26 | 1 | = |  |  |
| 71 | F | Distal | 45 | 2016 | pT2 | M0 | IIIA | + | 1,22 | 1,23 | 1 | = |  |  |
| 72 | M | Distal | 51 | 2015 | pT1(sm2) | M0 | IIIA | + | 1,73 | 2,11 | 22 | > |  |  |
| 73 | M | Distal | 79 | 2014 | pT2 | M0 | IIIA | + | 1,37 | 1,49 | 9 | = |  |  |
| 74 | F | Distal | 38 | 2016 | pT2 | M0 | IIIA | + | 1,39 | 1,43 | 3 | = |  |  |
| 75 | F | Proximal | 87 | 2010 | pT2 | M0 | IIIA | + | 1,53 | 1,83 | 20 | > |  |  |
| 76 | F | Distal | 75 | 2015 | pT2 | M0 | IIIA | + | 1,65 | 1,87 | 14 | > |  |  |
| 77 | M | Proximal | 70 | 2011 | pT3 | M0 | IIIB | + | 1,76 | 1,71 | -3 | = |  |  |
| 78 | F | Distal | 69 | 2015 | pT3 | M0 | IIIB | + | 0,00 | 1,82 |  |  |  |  |
| 79 | F | Distal | 86 | 2014 | pT3 | M0 | IIIB | + | 1,36 | 1,40 | 3 | = |  |  |
| 80 | F | Proximal | 85 | 2013 | pT3 | M0 | IIIB | + | 1,28 | 1,54 | 20 | > |  |  |
| 81 | M | Proximal | 63 | 2013 | pT3 | M0 | IIIB | + | 1,32 | 1,53 | 15 | > |  |  |
| 82 | F | Distal | 68 | 2016 | pT3 | M0 | IIIB | + | 1,33 | 1,30 | -2 | = |  |  |
| 83 | M | Distal | 87 | 2014 | pT3 | M0 | IIIB | + | 0,00 | 1,69 |  |  |  |  |
| 84 | M | Distal | 74 | 2014 | pT3 | M0 | IIIB | + | 1,38 | 1,32 | -4 | = |  |  |
| 85 | M | Proximal | 81 | 2014 | pT3 | M0 | IIIB | + | 1,33 | 1,65 | 24 | > |  |  |
| 86 | F | Proximal | 83 | 2013 | pT4a | M0 | IIIB | + | 1,41 | 1,47 | 5 | = |  |  |
| 87 | F | Distal | 74 | 2013 | pT3 | M0 | IIIB | + | 1,42 | 1,62 | 15 | > |  |  |
| 88 | M |  | 65 | 2016 | pT3 | M0 | IIIB | + | 1,23 | 1,20 | -3 | = |  |  |
| 89 | F |  | 87 | 2012 | pT3 | M0 | IIIB | + | 1,37 | 1,55 | 13 | > |  |  |
| 90 | M | Proximal | 66 | 2014 | pT3 | M0 | IIIB | + | 0,00 | 1,63 |  |  |  |  |
| 91 | F | Distal | 51 | 2013 | pT3 | M0 | IIIB | + | 1,22 | 1,23 | 1 | = |  |  |
| 92 | F | Proximal | 70 | 2013 | pT3 | M0 | IIIB | + | 1,42 | 1,80 | 27 | > |  |  |
| 93 | M | Proximal | 74 | 2016 | pT3 | M0 | IIIB | + | 1,66 | 1,82 | 10 | > |  |  |
| 94 | M | Distal | 81 | 2016 | pT3 | M0 | IIIB | + | 1,44 | 1,51 | 5 | = |  |  |
| 95 | F | Distal | 77 | 2014 | pT3 | M0 | IIIB | + | 1,75 | 1,94 | 11 | > |  |  |
| 96 | F | Distal | 76 | 2011 | pT3 | M0 | IIIB | + | 1,46 | 1,77 | 22 | > |  |  |
| 97 | F | Proximal | 78 | 2015 | pT3 | M0 | IIIB | + | 0,00 | 1,87 |  |  |  |  |
| 98 | F | Proximal | 91 | 2012 | pT3 | M0 | IIIB | + | 1,26 | 1,50 | 18 | > |  |  |
| 99 | F | Proximal | 67 | 2016 | pT3 | M0 | IIIB | + | 1,24 | 1,43 | 16 | > |  |  |
| 100 | F |  | 63 | 2013 | pT3 | M0 | IIIB | + | 0,00 | 1,40 |  |  |  |  |
| 101 | M | Proximal | 78 | 2012 | pT3 | M0 | IIIB | + | 1,27 | 1,18 | -7 | = |  |  |
| 102 | F | Proximal | 40 | 2012 | pT4a | M1 (HEP) | IIIB | + | 1,42 | 1,58 | 11 | > |  |  |
| 103 | M | Proximal | 60 | 2012 | pT4a | M0 | IIIC | + | 1,80 | 1,82 | 1 | = |  |  |
| 104 | F | Proximal | 68 | 2016 | pT3 | M0 | IIIC | + | 1,21 | 1,23 | 2 | = |  |  |
| 105 | M | Distal | 57 | 2014 | pT3 | M0 | IIIC | + | 1,67 | 1,79 | 7 | = |  |  |
| 106 | M | Proximal | 76 | 2011 | pT3 | M0 | IIIC | + | 1,29 | 1,60 | 24 | > |  |  |
| 107 | F | Distal | 71 | 2014 | pT3 | M0 | IIIC | + | 1,40 | 1,66 | 19 | > |  |  |
| 108 | F | Distal | 77 | 2014 | pT4b | M0 | IIIC | + | 1,37 | 1,29 | -6 | = |  |  |
| 109 | M | Distal | 65 | 2011 | pT3 | M0 | IIIC | + | 1,22 | 1,26 | 3 | = |  |  |
| 110 | F | Distal | 55 | 2012 | pT3/rT4a | M1 (PER/OTH) | IIIC | + | 0,00 | 1,19 |  |  |  |  |
| 111 | M | Proximal | 63 | 2011 | pT3 | M1 (HEP) | IV | + | 0,00 | 1,67 |  |  |  |  |
| 112 | F | Proximal | 41 | 2012 | pT3 | M1 (HEP) | IVA | + | 1,46 | 1,60 | 10 | > |  |  |
| 113 | M | Proximal | 79 | 2013 | pT3 | M1a | IVA | + | 1,50 | 1,67 | 11 | > |  |  |
| 114 | M | Distal | 81 | 2012 | pT4a | M1a (HEP) | IVA | + | 1,66 | 1,57 | -6 | = |  |  |
| 115 | F | Distal | 77 | 2015 | pT3 | M1a (HEP) | IVA | + | 1,53 | 1,77 | 16 | > |  |  |
| 116 | M | Proximal | 75 | 2013 | pT4b | M1a (HEP) | IVA | + | 1,14 | 1,12 | -2 | = |  |  |
| 117 | M | Proximal | 46 | 2012 | pT3 | M1a (HEP) | IVA | + | 1,22 | 1,76 | 44 | > |  |  |
| 118 | M | Proximal | 50 | 2016 | pT4b | M1a (HEP) | IVA | + | 1,31 | 1,36 | 4 | = |  |  |
| 119 | F | Proximal | 76 | 2015 | pT3 | M1a (HEP) | IVA | + | 1,78 | 1,72 | -3 | = |  |  |
| 120 | M |  | 83 | 2012 | pT3 | M1a (HEP) | IVA | + | 1,52 | 1,51 | -1 | = |  |  |
| 121 | M | Distal | 64 | 2013 | pT3 | M1a (OSS) | IVA | + | 1,26 | 1,22 | -3 | = |  |  |
| 122 | F | Proximal | 83 | 2016 | pT3 | M1b | IVB | + | 1,16 | 1,14 | -1 | = |  |  |
| 123 | M | Proximal | 85 | 2012 | pT3 | M1b (HEP) | IVB | + | 1,40 | 1,68 | 20 | > |  |  |
| 124 | M | Distal | 43 | 2013 | pT3 | M1b (LYM) | IVB | + | 0,00 | 1,20 |  |  |  |  |
| 125 | M | Proximal | 86 | 2012 | pT4b | M1b (PER) | IVB | + | 0,00 | 1,49 |  |  |  |  |
| 126 | M | Proximal | 79 | 2012 | pT3 | M1b (PER) | IVB | + | 1,54 | 1,44 | -6 | = |  |  |
| 127 | F | Proximal | 52 | 2013 | pT4a | M1b (PER) | IVB | + | 1,61 | 1,60 | -1 | = |  |  |
| 128 | M | Distal | 58 | 2014 | pT2 | M0 | I | + | 1,56 | 1,59 | 2 | = |  |  |
| 129 | M | Distal | 80 | 2015 | pT2 | M0 | I | + | 1,57 | 1,62 | 3 | = |  |  |
| 130 | F | Proximal | 63 | 2016 | pT2 | M0 | I | + | 1,66 | 1,60 | -3 | = |  |  |
| 131 | F | Proximal | 81 | 2016 | pT2 | M0 | I | + | 1,46 | 1,59 | 9 | = |  |  |
| 132 | M | Distal | 69 | 2016 | pT2 | M0 | I | + | 1,65 | 1,84 | 12 | > |  |  |
| 133 | F | Distal | 61 | 2016 | pT2 | M0 | I | + | 1,30 | 1,44 | 10 | > |  |  |
| 134 | F | Distal | 74 | 2014 | pT3 | M0 | II | + | 1,62 | 1,53 | -6 | = |  |  |
| 135 | M | Proximal | 57 | 2015 | pT3 | M0 | II | + | 1,43 | 1,68 | 17 | > |  |  |
| 136 | F | Distal | 46 | 2015 | pT3 | M0 | II | + | 1,36 | 1,54 | 13 | > |  |  |
| 137 | F | Proximal | 53 | 2016 | pT3 | M0 | II | + | 1,59 | 1,81 | 14 | > |  |  |
| 138 | F | Proximal | 71 | 2016 | pT3 | M0 | II | + | 1,43 | 1,42 | -1 | = |  |  |
| 139 | F | Distal | 46 | 2013 | pT3 | M1 (HEP) | IV | + | 1,61 | 1,54 | -4 | = |  |  |
| 140 | F | Distal | 74 | 2014 | pT3 | M1 (HEP) | IV | + | 1,37 | 1,28 | -6 | <^*^ |  |  |
| 141 | F | Proximal | 54 | 2016 | pT3 | M1 (HEP) | IV | + | 1,38 | 1,54 | 11 | > |  |  |
| 142 | F | Proximal | 58 | 2016 | pT3 | M1 (HEP) | IV | + | 1,49 | 1,69 | 14 | > |  |  |
| 143 | M | Proximal | 66 | 2013 | pT3 | M0 | IIA | + | 0,00 | 1,24 |  |  |  |  |
| 144 | M | Proximal | 84 | 2014 | pT3 | M0 | IIA | + | 1,48 | 1,74 | 17 | > |  |  |
| 145 | M | Proximal | 35 | 2016 | pT3 | M0 | IIA | + | 1,32 | 1,53 | 16 | > |  |  |
| 146 | M | Proximal | 40 | 2013 | pT3 | M0 | IIIB | + | 1,35 | 1,40 | 4 | = |  |  |
| 147 | F | Distal | 85 | 2011 | pT3 | M0 | IIIB | + | 0,00 | 1,63 |  |  |  |  |
| 148 | F | Proximal | 71 | 2011 | pT3 | M1 (PUL) | IIIB | + | 1,45 | 1,59 | 10 | > |  |  |
| 149 | F | Proximal | 73 | 2016 | pT3 | M0 | IIIC | + | 0,00 | 1,24 |  |  |  |  |
| 150 | M | Proximal | 68 | 2015 | pT4 | M1 | IVA | + | 0,00 | 1,98 |  |  |  |  |
| 151 | F | Proximal | 79 | 2016 | pT4b | M1a | IVA | + | 1,15 | 1,46 | 27 | > |  |  |
| 152 | F | Proximal | 65 | 2015 | pT3 | M1a (HEP) | IVA | + | 1,59 | 1,69 | 6 | = |  |  |
| 153 | M |  | 56 | 2011 | pT3 | M1a (HEP) | IVA | + | 1,51 | 1,62 | 7 | = |  |  |
| 154 | M | Proximal | 70 | 2015 | pT4a | M1b | IVB | + | 1,50 | 1,65 | 10 | > |  |  |
| 155 | M | Distal | 62 | 2013 | pT4b | M1b (LYM) | IVB | + | 1,90 | 1,39 | -27 | < |  |  |
| 156 | F | Distal | 52 | 2013 | pT4a | M1 (HEP) | IV | + | 1,49 | 1,57 | 5 | = |  |  |
| 157 | F | Proximal | 48 | 2013 | pT4b | M1 (HEP) | IV | + | 1,45 | 1,46 | 1 | = |  |  |
| 158 | F | Proximal | 65 | 2014 | pT3 | M1 (HEP) | IV | + | 1,31 | 1,30 | -1 | = |  |  |
| 159 | M | Proximal | 74 | 2016 | pT3 | M0 | II | + | 1,50 | 1,78 | 19 | > |  |  |
| 160 | F | Proximal | 48 | 2014 | pT3 | M0 | IIIC | + | 1,81 | 1,64 | -9 | = |  |  |
| 161 | F | Proximal | 48 | 2012 | pT3 | M1b (PER) | IVB | + | 1,47 | 1,46 | 0 | = |  |  |
| 162 | M | Proximal | 62 | 2014 | pT2 | M1b (OTH) | IVB | + | 1,32 | 1,28 | -3 | = |  |  |
| 163 | F | Distal | 66 | 2014 | pT3 | M0 | I | - | 0,00 | 1,68 |  |  | + | BRAF wt |
| 164 | F | Proximal | 85 | 2016 | pT1 | M0 | I | - | 1,48 | 1,33 | -10 | < | + | BRAF wt |
| 165 | F | Proximal | 79 | 2013 | pT3 | M0 | IIA | - | 0,00 | 1,55 |  |  | + | BRAF V600^E/E2/D^ |
| 166 | F | Proximal | 68 | 2014 | pT2 | M0 | I | - | 1,70 | 1,63 | -4 | = | + | BRAF V600^E/E2/D^ |
| 167 | F |  | 73 | 2013 | pT2 | M0 | I | - | 1,32 | 1,28 | -3 | = | + | BRAF V600^E/E2/D^ |
| 168 | M | Proximal | 84 | 2015 | pT1(sm3) | M0 | I | - | 1,52 | 1,70 | 12 | > | + | BRAF wt |
| 169 | M |  | 72 | 2015 | pT2 | M0 | I | - | 1,76 | 1,77 | 0 | = | + | BRAF wt |
| 170 | F | Proximal | 94 | 2014 | pT4b | M0 | IIC | - | 1,72 | 1,47 | -15 | < | + | BRAF V600^E/E2/D^ |
| 171 | F | Proximal | 75 | 2013 | pT3 | M0 | IIIB | - | 1,29 | 1,38 | 7 | = | + | BRAF V600^E/E2/D^ |
| 172 | F | Proximal | 70 | 2014 | pT3 | M0 | IIIB | - | 1,61 | 1,79 | 11 | > | + | BRAF V600^E/E2/D^ |
| 173 | M | Proximal | 75 | 2014 | pT4b | M1a (LYM) | IVA | - | 1,40 | 1,09 | -22 | < | -^**^ | BRAF wt |
| 174 | F | Proximal | 44 | 2016 | pT3 | M1b | IVB | - | 0,00 | 1,53 |  |  | -^**^ | BRAF wt |
| 175 | F | Proximal | 75 | 2016 | pT3 | M1b (HEP, LYM) | IVB | - | 1,40 | 1,38 | -1 | = | + | BRAF V600^E/E2/D^ |
| 176 | M | Distal | 38 | 2006 | pT4 | M0 | II | - | 1,51 | 1,54 | 2 | = | + | BRAF wt |
| 177 | M | Distal | 29 | 2008 | pT3c | M0 | II | - | 1,45 | 1,24 | -14 | < | + | BRAF wt |
| 178 | F | Distal | 40 | 2012 | pT3 | M0 | II | - | 1,22 | 1,73 | 41 | > | + | BRAF wt |
| 179 | M | Proximal | 71 | 2016 | pT3 | M0 | II | - | 1,72 | 1,41 | -18 | < | -^**^ | BRAF wt |
| 180 | M | Distal | 47 | 2013 | pT3 | M1 (HEP) | IV | - | 1,75 | 1,38 | -22 | < | -^**^ | BRAF wt |
| 181 | F | Proximal | 76 | 2015 | pT3 | M1 (HEP) | IV | - | 1,58 | 1,81 | 14 | > | + | BRAF V600^E/E2/D^ |
| 182 | F | Proximal | 82 | 2012 | pT4b | M1a | IVA | - | 1,22 | 1,42 | 17 | > | -^**^ | BRAF wt |
| 183 | M |  | 46 | 2012 | pT4b | M0 | II | - | 1,53 | 1,37 | -10 | < | + | BRAF wt |
| 184 | F |  | 66 | 2013 | pT3 | M0 | II | - | 1,36 | 1,38 | 2 | = | + | BRAF V600^E/E2/D^ |
| 185 | M | Proximal | 41 | 2015 | pT4b | M0 | II | - | 1,77 | 1,66 | -6 | = | + | BRAF wt |
| 186 | M |  | 64 | 2014 | pT4b | M0 | III | - | 1,69 | 1,31 | -22 | < | + | BRAF V600^E/E2/D^ |
| 187 | F | Proximal | 65 | 2016 | pT3 | M0 | II | - | 1,51 | 1,75 | 16 | > | + | BRAF wt |
| 188 | F | Proximal | 74 | 2016 | pT3 | M0 | II | - | 1,53 | 1,30 | -15 | < | + | BRAF V600^E/E2/D^ |
| 189 | F | Proximal | 77 | 2016 | pT4a | M0 | IVA | - | 1,65 | 1,75 | 6 | = | + | BRAF V600^E/E2/D^ |

^1)^ M = male; F = female;

^2)^ HEP = liver; PUL = lung; PER = peritoneal; OSS = bones; LYM = distant lymph nodes; OTH = other

^*)^ Cases required readjustments due to high background signals

^**)^ no MSI detectable with Idylla system
