# Supplementary material for: Downregulation of SPTAN1 is related to MLH1 deficiency and metastasis in colorectal cancer
Source: PLoS One. 2019 Mar 11;14(3):e0213411. doi: 10.1371/journal.pone.0213411 (PMC6411122; doi:10.1371/journal.pone.0213411)
Supplement: S2 Table — (DOCX) [file pone.0213411.s005.docx]

**S2 Table. Comparison of SPTAN1 expression**

|  | **All  (n=152)** | **MLH1 positive (n=128)** | **MLH1 negative (n=24)** | **p-value^1^** |
| --- | --- | --- | --- | --- |
| Expression compared to mucosa  SPTAN1 ↓ n (%)  SPTAN1 ↔ or ↑ n (%) | 14 (9.2%) 138 (90.8%) | 5 (3.9%) 123 (96.1%) | 9 (37.5%) 15 (62.5%) | <0.001 |

^1^ p-values from comparisons between MLH1-positive and MLH1-negative patients, determined by Fisher’s exact test
